# Supplementary material for: Phosphorylation-mediated conformational change regulates human SLFN11
Source: Nat Commun. 2024 Dec 3;15:10500. doi: 10.1038/s41467-024-54833-7 (PMC11615386; doi:10.1038/s41467-024-54833-7)
Supplement: Supplementary file 1 — Supplementary Information [file 41467_2024_54833_MOESM1_ESM.pdf]

Supplementary Information

Phosphorylation-mediated conformational change regulates human SLFN11

Michael Kugler, Felix J. Metzner, Gregor Witte, Karl-Peter Hopfner and Katja Lammens

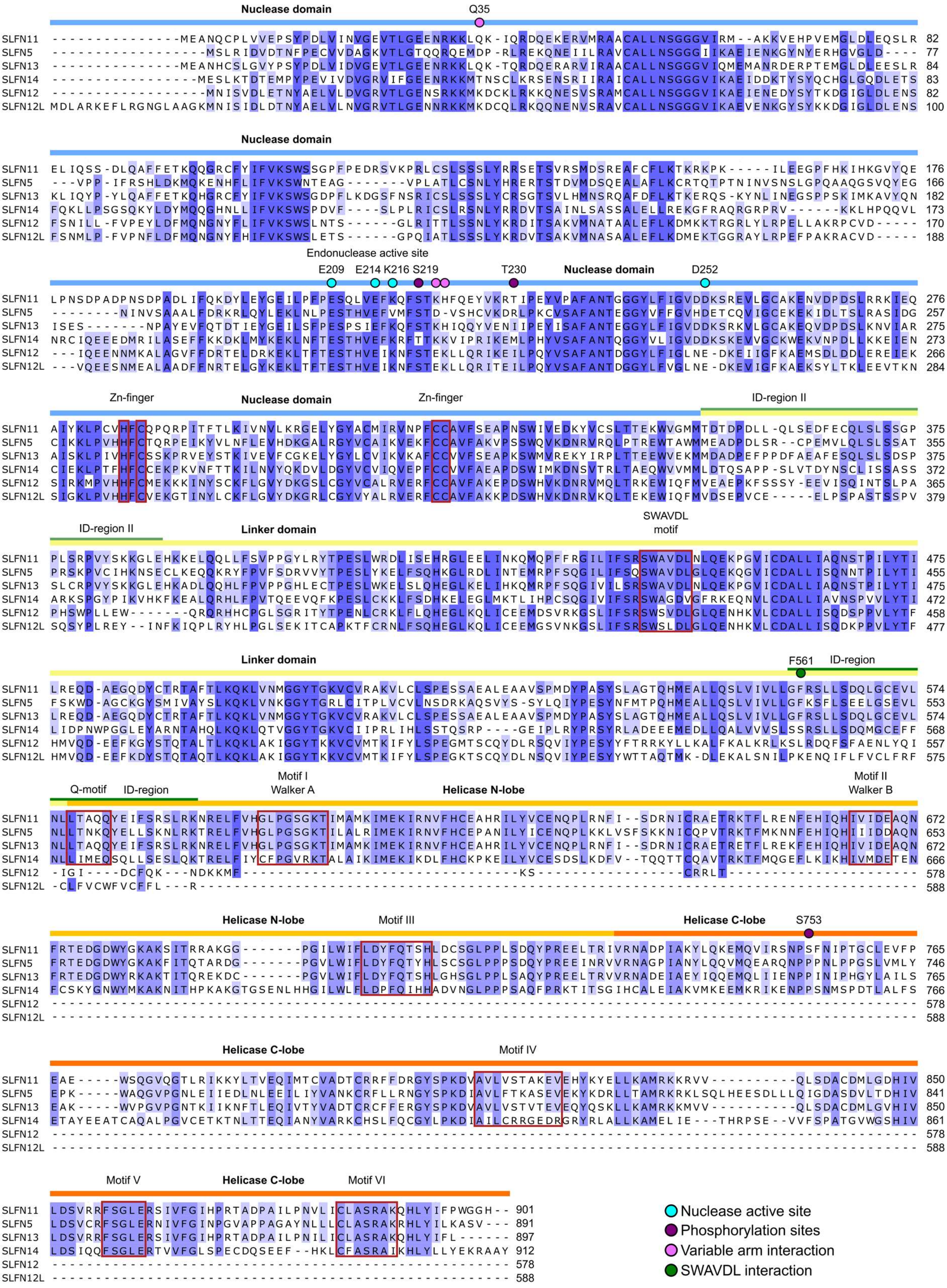

Supplementary Fig. 1: Multiple sequence alignment of SLFN11 with human Slfn proteins. Sequence alignment of human (SLFN11, SLFN5, SLFN13, SLFN14, SLFN12, SLFN12L) Slfn protein sequences was calculated using Clustal Omega[1]. Individual residues

are colored according to percentage identity (dark blue: more conserved, white: less conserved). The nuclease domain is indicated in blue, the linker domain in yellow, the helicase domain in orange and the ID and ID II regions in green and light green, respectively. The zinc finger, the SWAVDL motif and the helicase motifs are highlighted with red boxes. The nuclease active site residues (cyan), phosphorylation sites (purple), variable arm interactions (pink) and a SWAVDL interaction (green) are highlighted with colored circles.

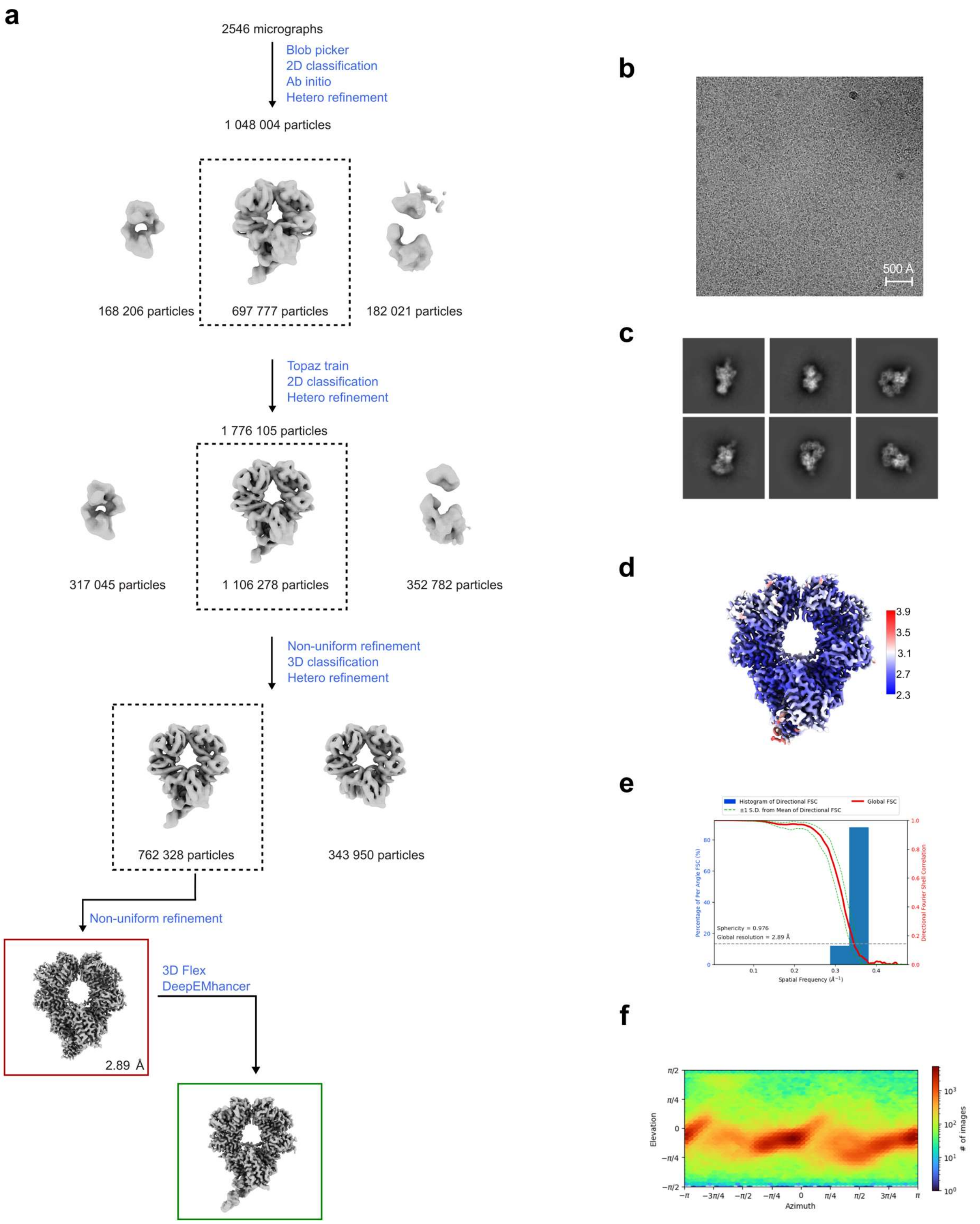

Supplementary Fig. 2: Cryo-EM data analysis of SLFN11^{wt} with tRNA-Leu-TAA. a, Cryo-EM data processing workflow of SLFN11^{wt} with tRNA-Leu using cryoSPARC[2]. **b**, Representative micrograph of SLFN11^{wt} with tRNA-Leu. The displayed micrograph is representative of 2,546 movies collected. **c**, Representative classes of a 2D classification of the particles used for the final SLFN11^{wt} with tRNA-Leu reconstruction. **d**, Visualization of local resolution of SLFN11^{wt} with tRNA-Leu calculated in cryoSPARC. Blue indicates higher

resolution and red indicates lower resolution. **e**, Histogram of directional FSC[3] (blue) and global FSC curve (red) of the final SLFN11^{wt} with tRNA-Leu reconstruction. The spread of directional resolution values is defined as ±1σ (dashed green lines). The grey dashed line indicates the 0.143 cutoff criterion, indicating a nominal resolution of 2.89 Å. **f**, Angular distribution of the particles used for the final SLFN11^{wt} with tRNA-Leu reconstruction.

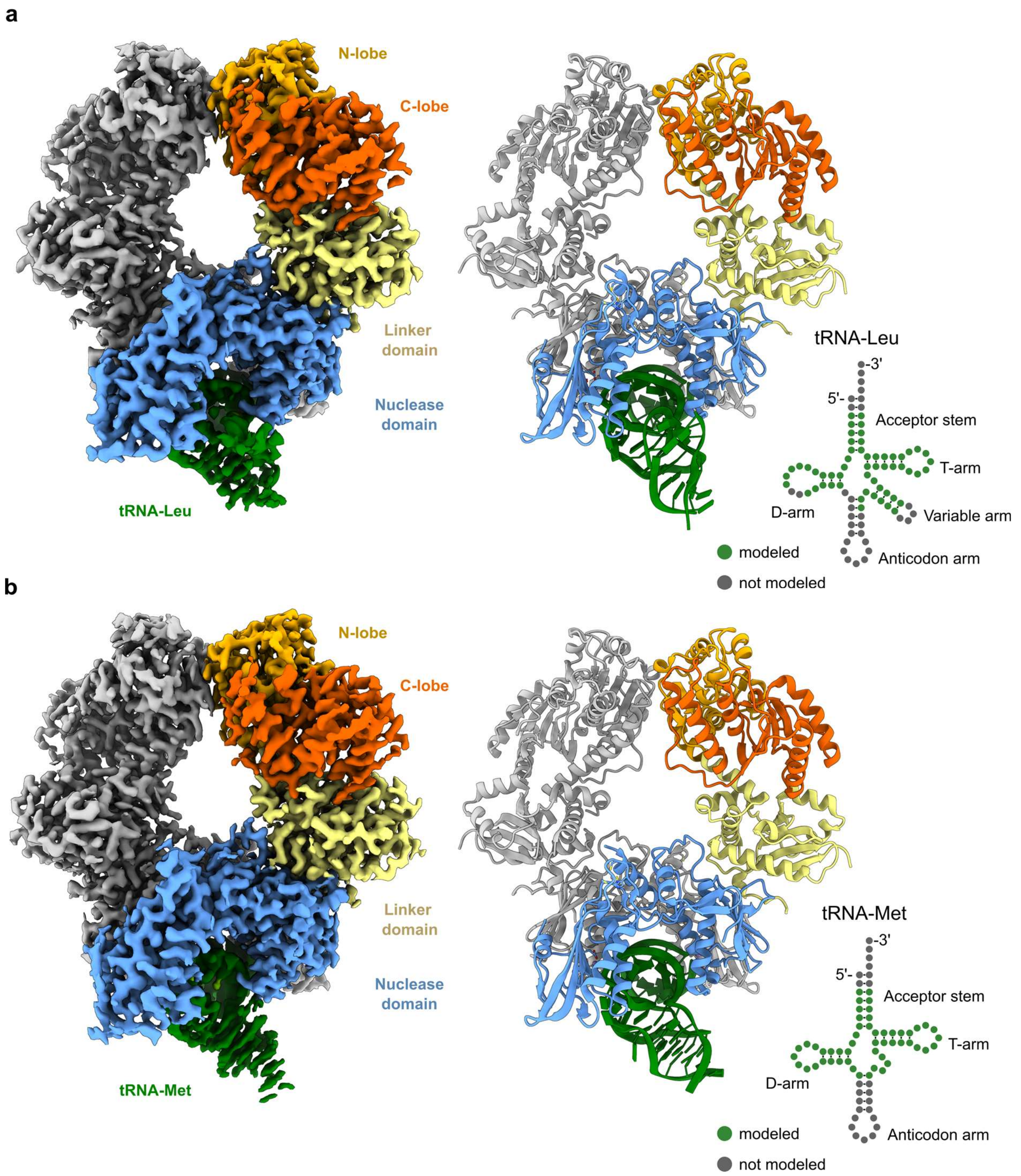

Supplementary Fig. 3: Overview of SLFN11 tRNA bound structures. Cryo-EM map (left) and cartoon representation (right) of SLFN11 bound to tRNA-Leu **a** and tRNA-Met **b**. Schematic representations of tRNA-Leu **a** and tRNA-Met **b**. Nucleotides that are modeled in the structure are depicted in green, while nucleotides that are not modeled in the structure are depicted in grey.

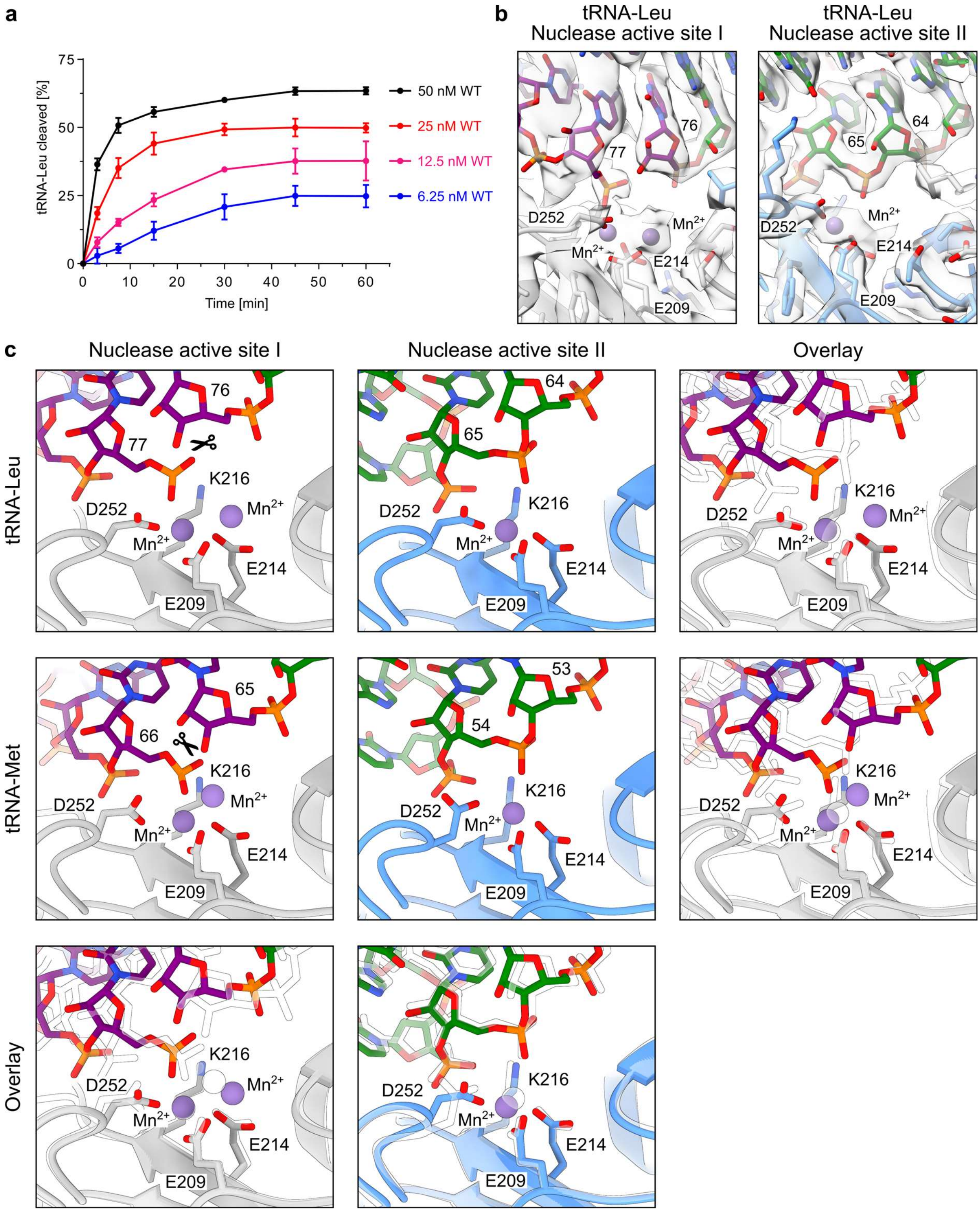

Supplementary Fig. 4: Structural comparison of nuclease active sites of SLFN11 tRNA-Leu and tRNA-Met bound structures. a, Analysis of the endonucleolytic cleavage of tRNA-Leu at various SLFN11^{wt} concentrations. Data are represented as mean values +/− SD from three independent experiments. **b**, Close-up views of SLFN11 nuclease active sites I and II for the tRNA-Leu bound structure. The semitransparent cryo-EM map is shown together with the model. Nuclease active site residues (E209, E214, D252) and the manganese ions are labeled.

c, Close-up views of SLFN11 nuclease active sites I and II for the tRNA-Leu and tRNA-Met bound structures, respectively. The nuclease active site residues (E209, E214, K216, D252) and the manganese ions are shown. The tRNA cleavage site is indicated in nuclease active sites I. Structural overlays of the nuclease active sites I and II for each tRNA and overlays of the nuclease active sites I or II for the different tRNAs are shown. Source data for **a** are provided as a Source Data file.

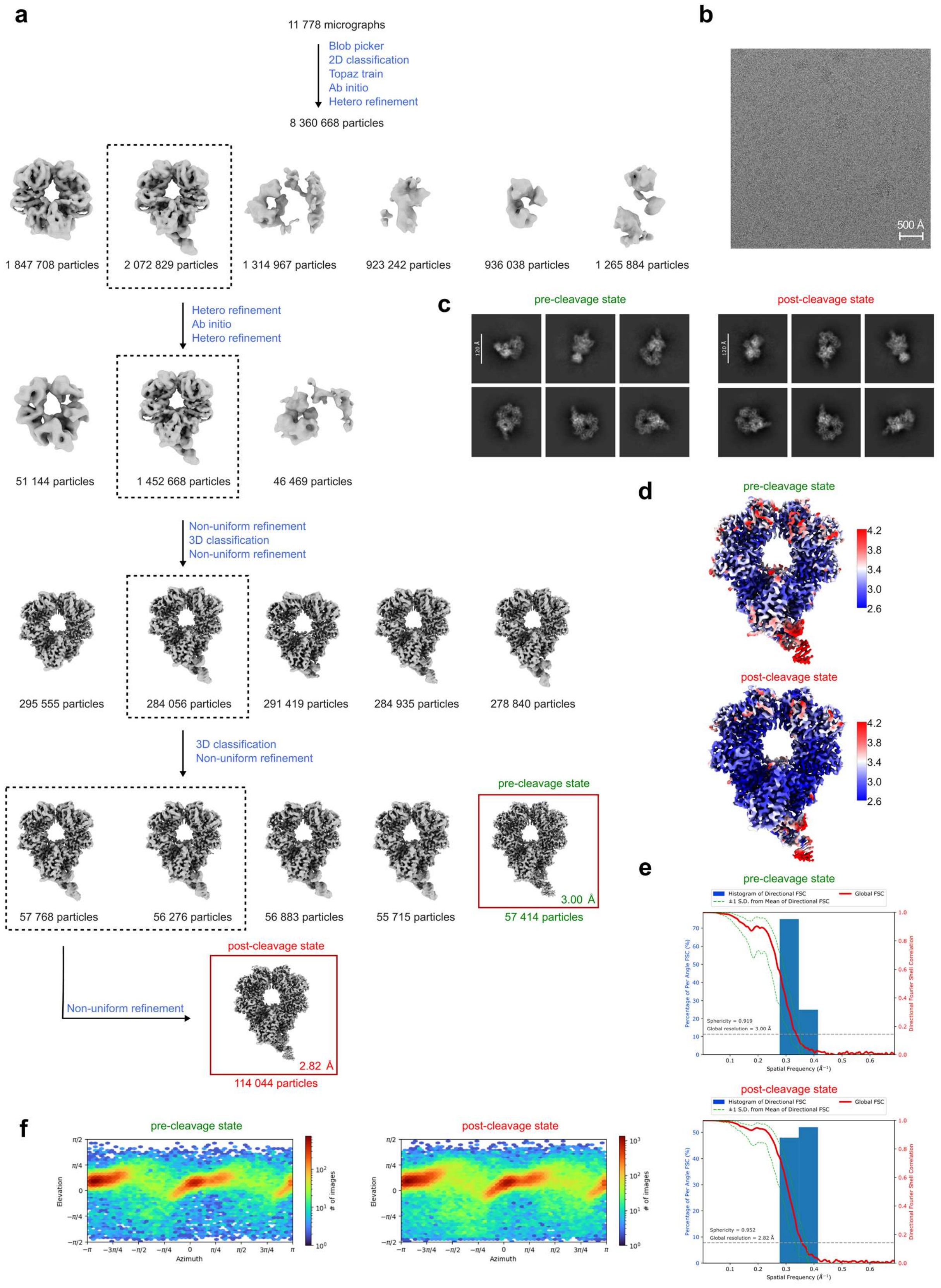

Supplementary Fig. 5: Cryo-EM data analysis of SLFN11^{wt} with tRNA-Leu-TAA in pre-cleavage and post-cleavage state. a, Cryo-EM data processing workflow of SLFN11^{wt} with tRNA-Leu using cryoSPARC[2]. Final reconstructions of pre-cleavage and post-cleavage states

are highlighted in green and red, respectively. **b**, Representative micrograph of $SLFN11^{wt}$ with tRNA-Leu. The displayed micrograph is representative of 11,778 movies collected. **c**, Representative classes of a 2D classification of the particles used for the final $SLFN11^{wt}$ with tRNA-Leu reconstruction in pre-cleavage and post-cleavage state. **d**, Visualization of local resolution of $SLFN11^{wt}$ with tRNA-Leu in pre-cleavage and post-cleavage state calculated in cryoSPARC. Blue indicates higher resolution and red indicates lower resolution. **e**, Histogram of directional FSC[3] (blue) and global FSC curve (red) of the final $SLFN11^{wt}$ with tRNA-Leu reconstruction in pre-cleavage and post-cleavage state. The spread of directional resolution values is defined as $\pm 1\sigma$ (dashed green lines). The grey dashed line indicates the 0.143 cutoff criterion, indicating a nominal resolution of 3.00 Å and 2.82 Å for the pre-cleavage and post-cleavage state, respectively. **f**, Angular distribution of the particles used for the final $SLFN11^{wt}$ with tRNA-Leu reconstruction in pre-cleavage and post-cleavage state.

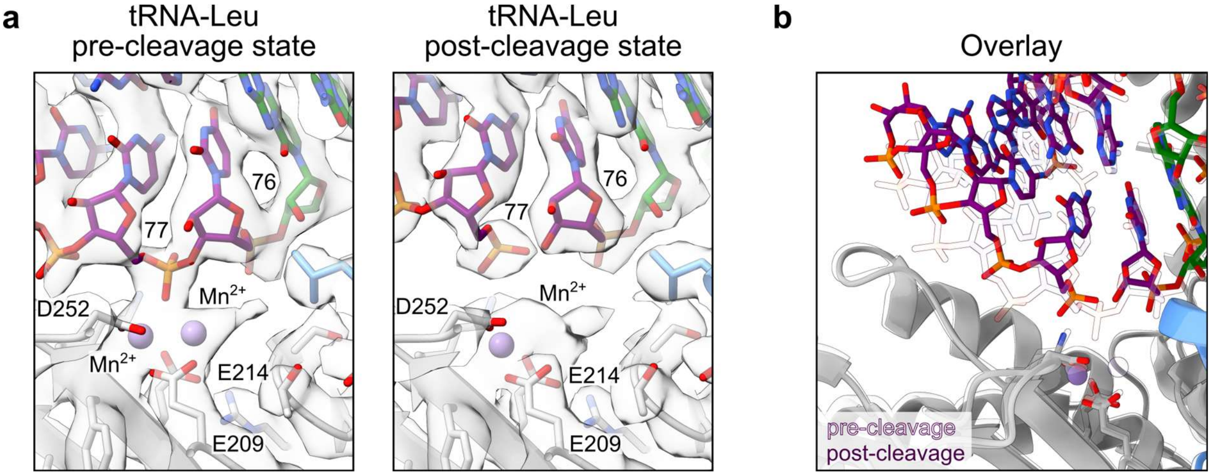

Supplementary Fig. 6: Structural comparison of SLFN11 bound tRNA-Leu pre- and post-cleavage states. a, Close-up views of SLFN11 nuclease active site I for the tRNA-Leu pre- and post-cleavage states. The semitransparent cryo-EM maps are shown together with the models. Nuclease active site residues (E209, E214, D252) and the manganese ions are labeled. **b**, Structural comparison of tRNA-Leu pre- and post-cleavage states at nuclease active site I. The pre-cleavage state is shown semitransparent.

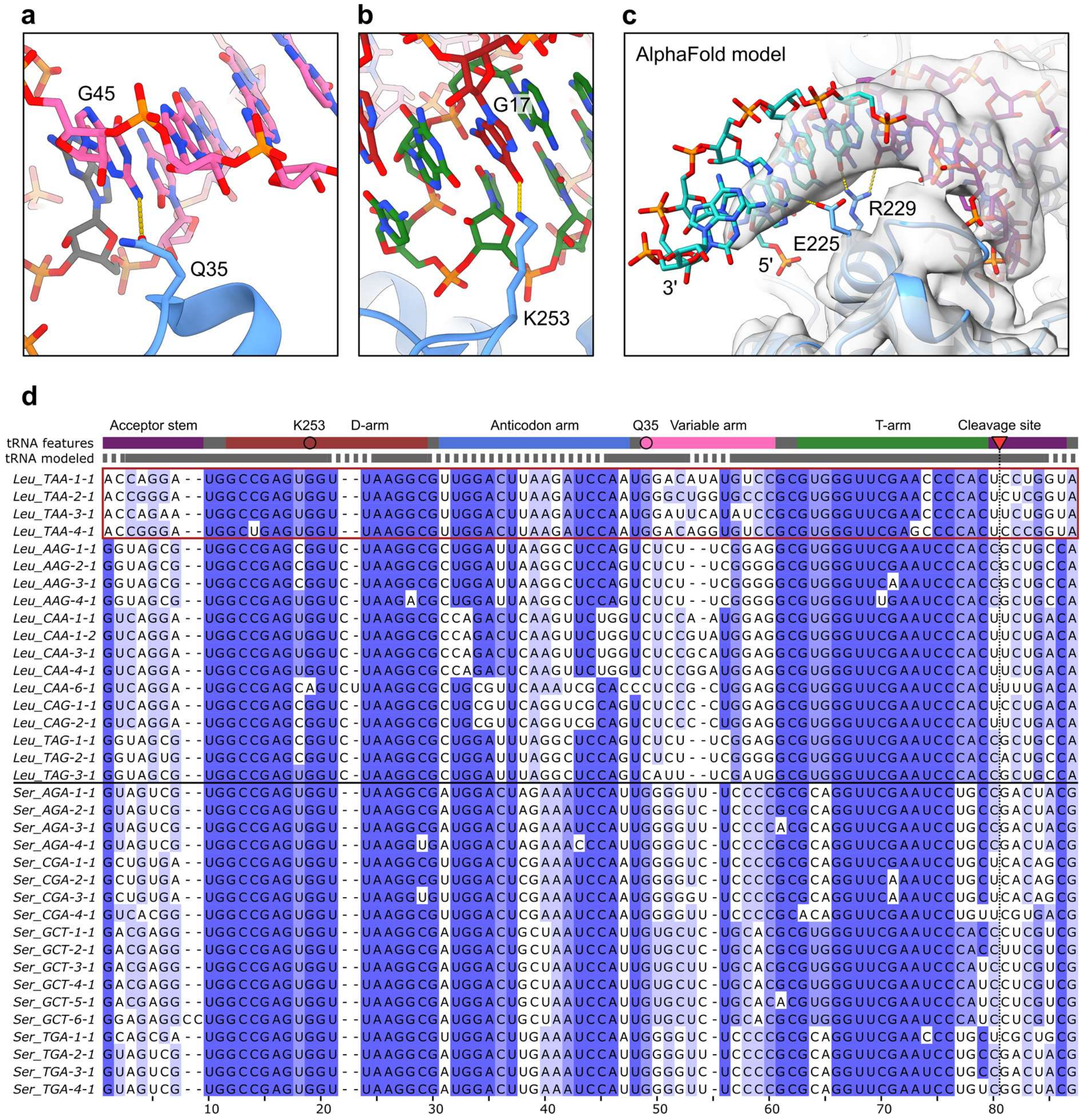

Supplementary Fig. 7: SLFN11-tRNA interaction. **a**, Detailed view of the interaction between SLFN11 Q35 and G45 of the tRNA-Leu-TAA variable arm. **b**, Detailed view of the interaction between SLFN11 K253 and G17 of the tRNA-Leu-TAA D-arm. **c**, An AlphaFold[4] model of tRNA-Leu-TAA bound to SLFN11 is rigid body docked into the unsharpened cryo-EM map of SLFN11 bound to tRNA-Leu-TAA. The region of the tRNA that is also present in the experimental structure is colored in purple, while the part that is just present in the AlphaFold model is colored in teal. The AlphaFold model suggests sequence read out of several bases of the tRNA acceptor stem by SLFN11 residues E225 and R229. **d**, Multiple sequence alignment of Leu and Ser type II tRNAs. The sequence alignment was calculated using Mafft[5]. Individual bases are colored according to percentage identity (dark blue: more conserved, white: less conserved). tRNA-Leu-TAA sequences are highlighted with a red box. The

structural features of the tRNA (acceptor stem: purple, D-arm: red, anticodon arm: blue, variable arm: pink: T-arm: green) and the region of tRNA-Leu that is modeled in SLFN11-tRNA-Leu structure (gray line: modeled, dotted line: not modeled) are indicated. SLFN11 residues that directly interact with tRNA bases are indicated with colored circles. The SLFN11 cleavage site is indicated by a red triangle.

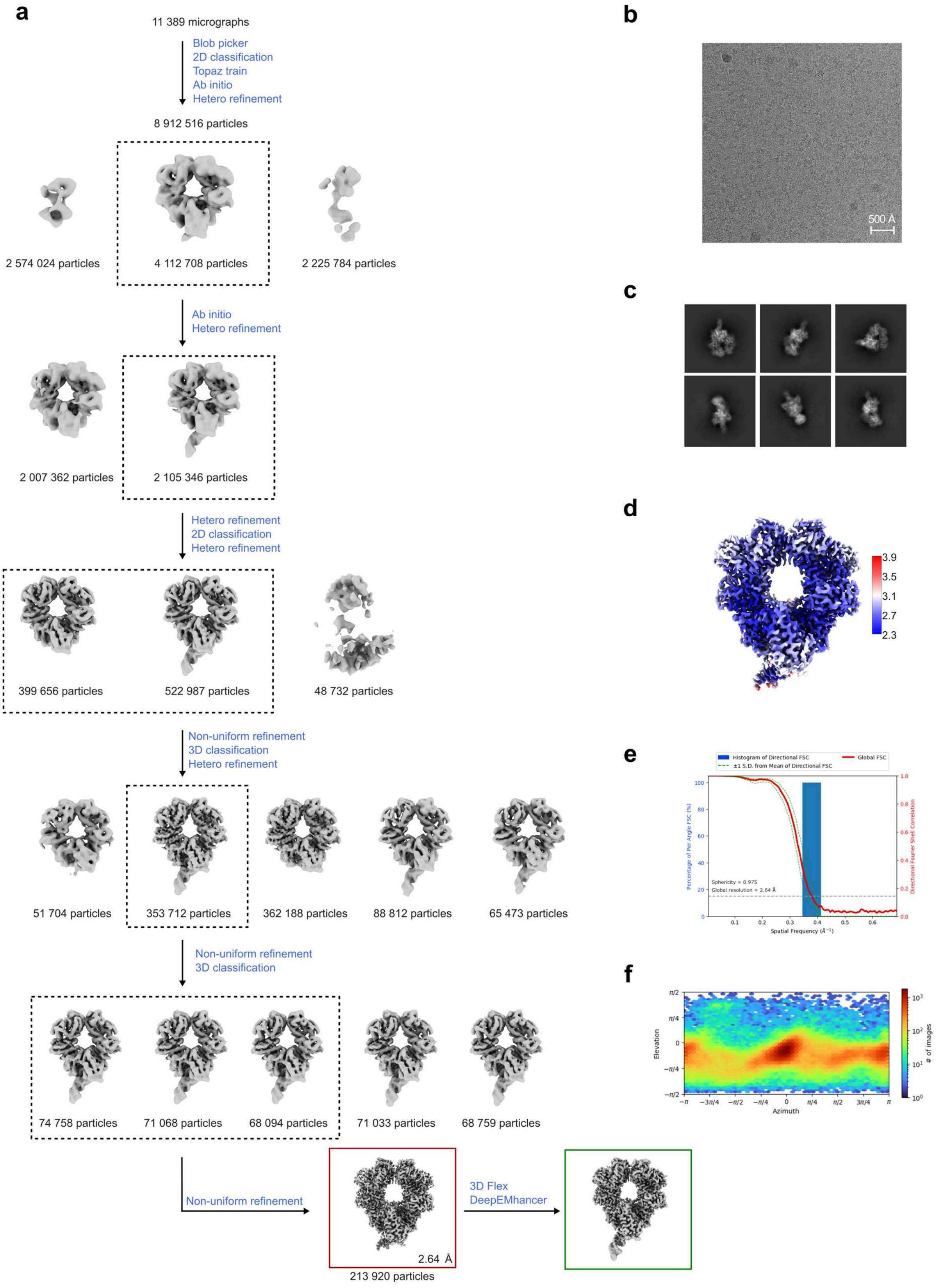

Supplementary Fig. 8: Cryo-EM data analysis of SLFN11^{wt} with tRNA-Met-CAT. a, Cryo-EM data processing workflow of SLFN11^{wt} with tRNA-Met using cryoSPARC[2]. **b**, Representative micrograph of SLFN11^{wt} with tRNA-Met. The displayed micrograph is

representative of 11,389 movies collected. **c**, Representative classes of a 2D classification of the particles used for the final SLFN11^{wt} with tRNA-Met reconstruction. **d**, Visualization of local resolution of SLFN11^{wt} with tRNA-Met calculated in cryoSPARC. Blue indicates higher resolution and red indicates lower resolution. **e**, Histogram of directional FSC[3] (blue) and global FSC curve (red) of the final SLFN11^{wt} with tRNA-Met reconstruction. The spread of directional resolution values is defined as ±1σ (dashed green lines). The grey dashed line indicates the 0.143 cutoff criterion, indicating a nominal resolution of 2.64 Å. **f**, Angular distribution of the particles used for the final SLFN11^{wt} with tRNA-Leu reconstruction.

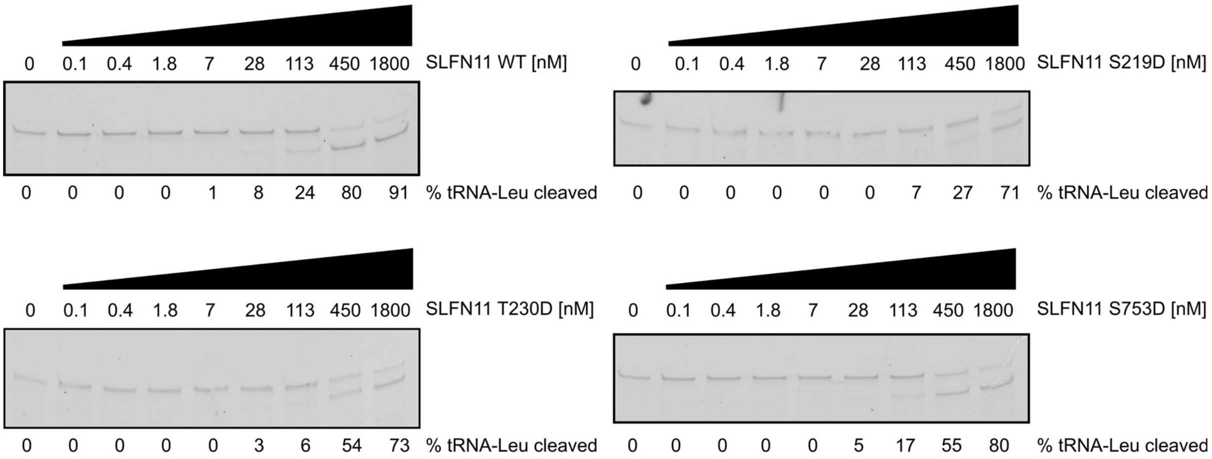

Supplementary Fig. 9: Analysis of the SLFN11 endonucleolytic cleavage of tRNA-Leu at conditions used in MST experiments. Effect of phosphomimetic mutations on cleavage of tRNA-Leu monitored by nuclease assay. Source data are provided as a Source Data file.

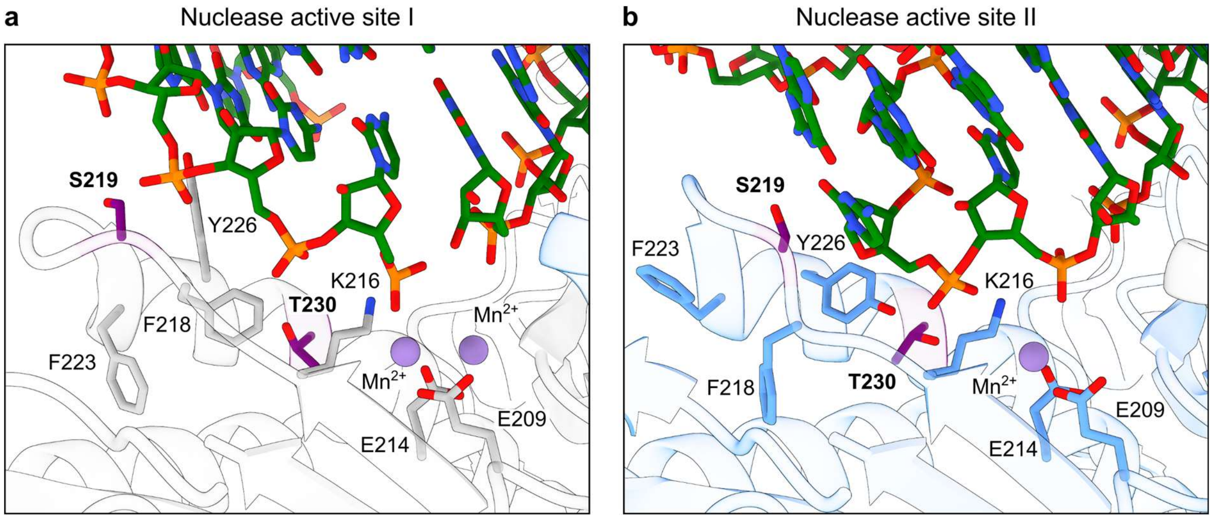

Supplementary Fig. 10: Detailed view of the phosphorylation sites within the nuclease domain of SLFN11. Positions of phosphorylation sites S219 and T230 (purple) in the proximity to nuclease active sites I **a** and II **b** in the tRNA-Leu bound SLFN11 structure. tRNA-Leu is depicted in green. The side chains of the phosphorylation sites (S219, T230) (purple), nuclease active site (E209, E214, and K216) and three adjacent aromatic residues that undergo a conformational change (F218, F223, and Y226) are shown.

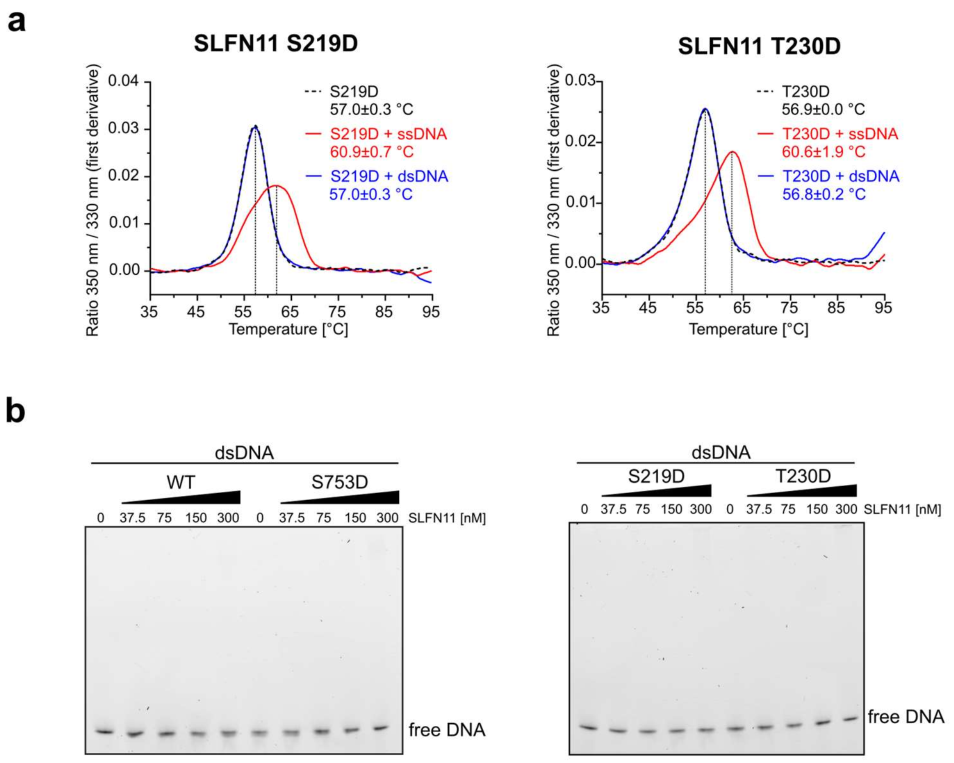

Supplementary Fig. 11: DNA binding of SLFN11 phosphomimetic mutants. a, NanoDSF measurements of SLFN11^{S219D} (left) and SLFN11^{T230D} (right) in the presence of ssDNA or dsDNA. Data are represented as mean values +/− SD from three independent experiments. One representative replicate is shown. **b**, dsDNA binding of SLFN11^{wt}, SLFN11^{S753D} (left), and SLFN11^{S219D}, SLFN11^{T230D} (right) monitored by electrophoretic mobility shift assay. The experiment was performed in duplicates. One representative replicate is shown. Source data for **a** and **b** are provided as a Source Data file.

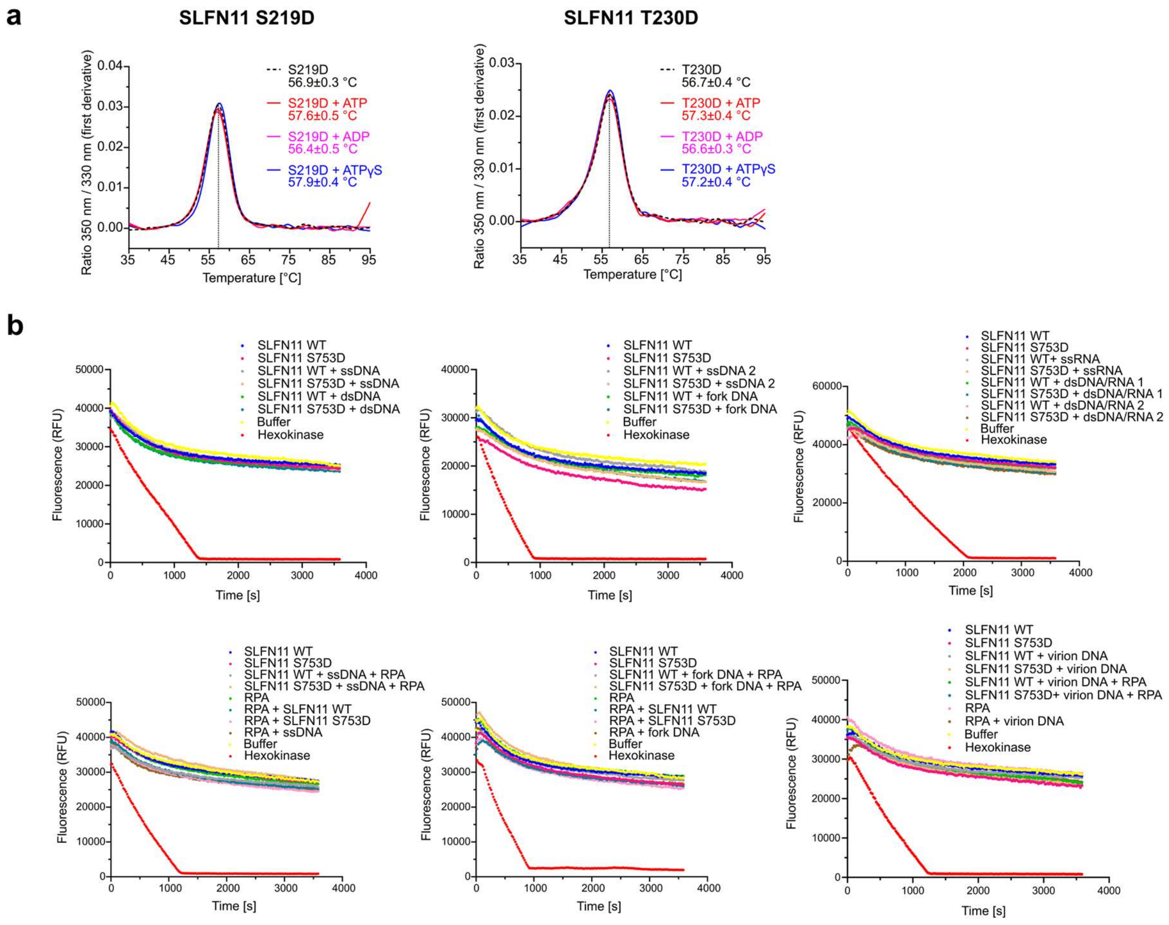

Supplementary Fig. 12: Nucleotide binding and ATPase activity of SLFN11 phosphomimetic mutants. **a**, NanoDSF measurements of SLFN11^{S219D} (left) and SLFN11^{T230D} (right) without nucleotide or in presence of different nucleotides. Data are represented as mean values +/− SD from three independent experiments. One representative replicate is shown. **b**, A fluorescence-based assay was employed to measure the ATPase activity of SLFN11^{wt} and SLFN11^{S753D}, with buffer only as a negative control and hexokinase as a positive control. The fluorescence of NADH is monitored over time. ATP consumption leads to NADH oxidation, resulting in decreased fluorescence due to ATP hydrolysis. The details of individual substrates are shown in Supplementary Table 2. Source data for **a** and **b** are provided as a Source Data file.

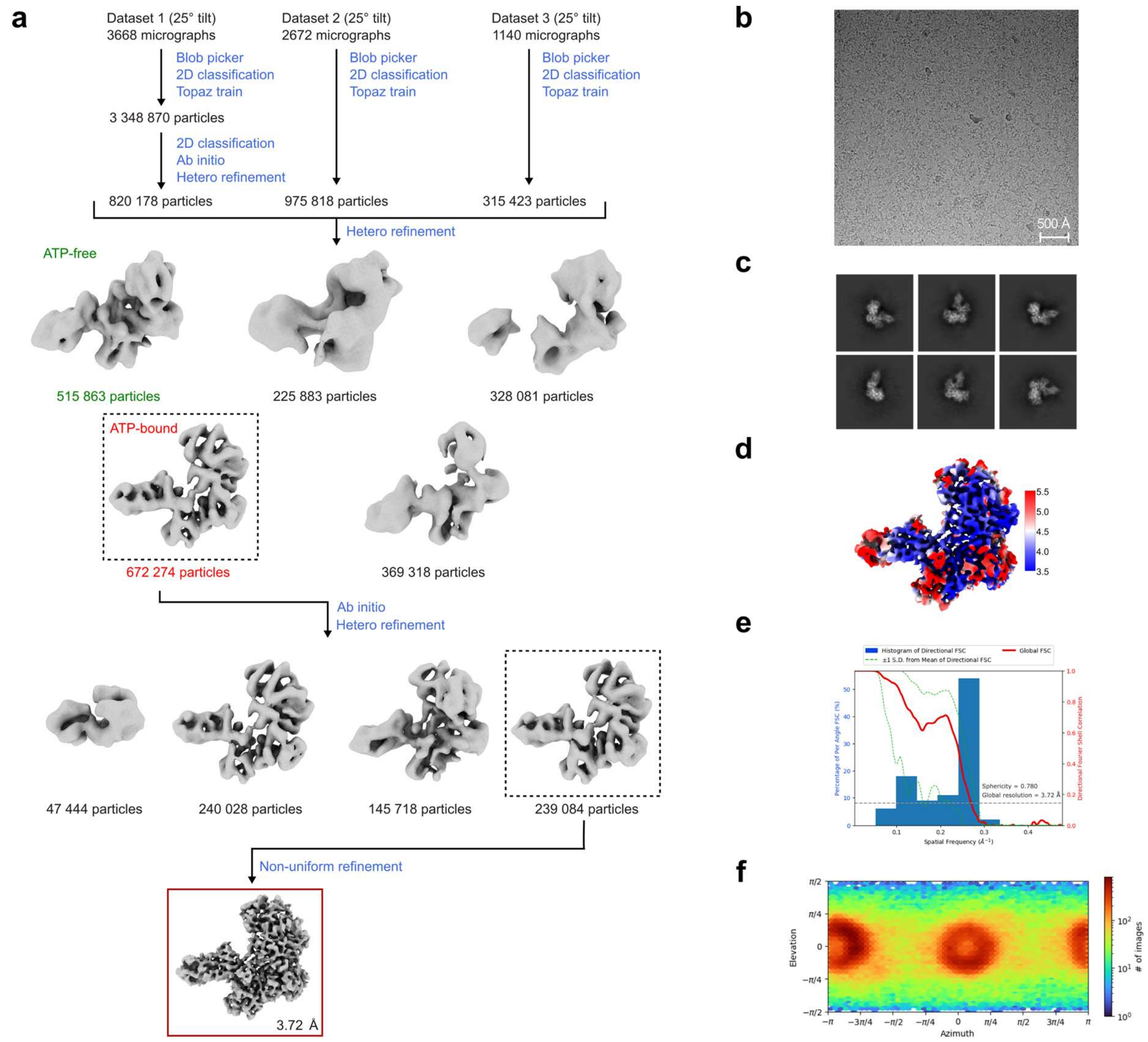

Supplementary Fig. 13: Cryo-EM data analysis of SLFN11^{S753D}. a, Cryo-EM data processing workflow of SLFN11^{S753D} using cryoSPARC[2]. **b**, Representative micrograph of SLFN11^{S753D}. The displayed micrograph is representative of 7,480 movies collected. **c**, Representative classes of a 2D classification of the particles used for the final SLFN11^{S753D} reconstruction. **d**, Visualization of local resolution of SLFN11^{S753D} calculated in cryoSPARC. Blue indicates higher resolution and red indicates lower resolution. **e**, Histogram of directional FSC[3] (blue) and global FSC curve (red) of the final SLFN11^{S753D} reconstruction. The spread of directional resolution values is defined as ±1σ (dashed green lines). The grey dashed line indicates the 0.143 cutoff criterion, indicating a nominal resolution of 3.72 Å. **f**, Angular distribution of the particles used for the final SLFN11^{S753D} reconstruction.

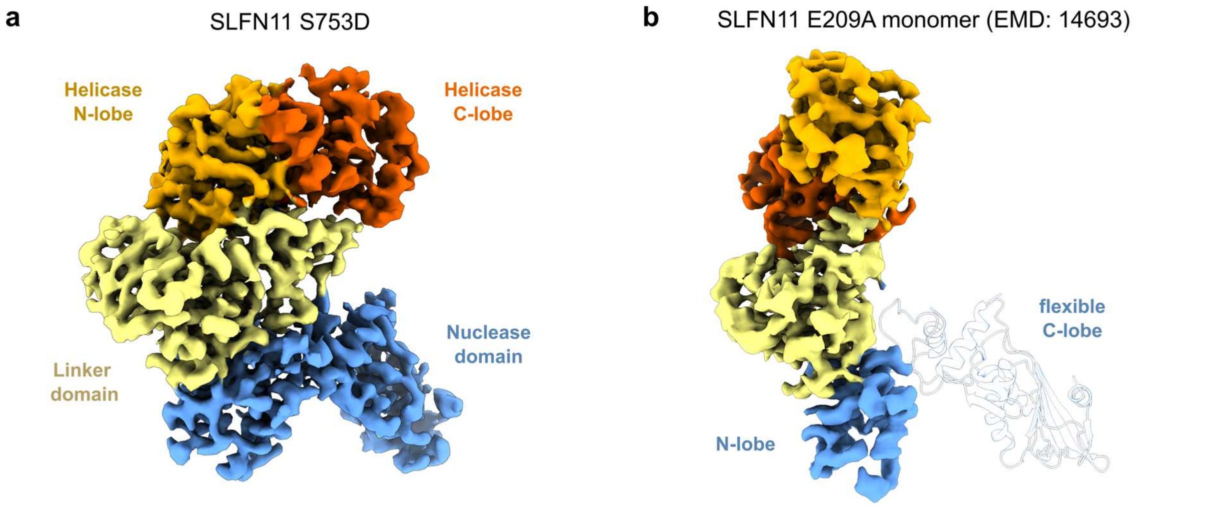

Supplementary Fig. 14: Conformational differences of the SLFN11^{S753D} and SLFN11^{E209A} monomers. a, Cryo-EM reconstruction of SLFN11^{S753D}. The protein domains are color-coded (nuclease domain: blue, linker domain: yellow, helicase N-lobe: light orange, helicase C-lobe: dark orange) and labelled. **b**, Cryo-EM reconstruction of monomeric SLFN11^{E209A} (EMD: 14693). The protein domains are color-coded according to **a**. The position of the flexible C-lobe of the nuclease domain is depicted as a transparent cartoon model based on the structure of dimeric SLFN11^{wt} (PDB: 7ZEL).

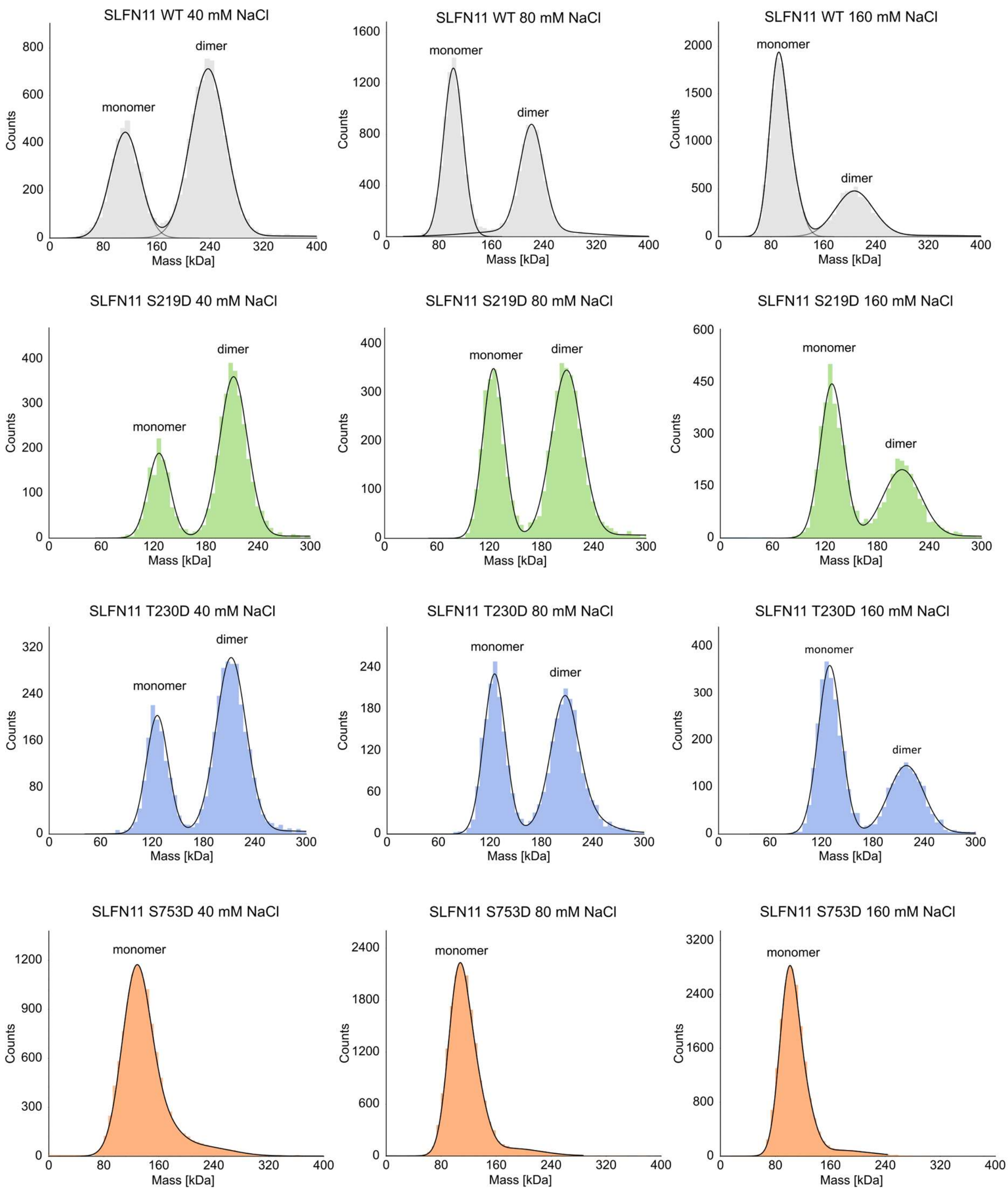

Supplementary Fig. 15: Analysis of oligomeric state of SLFN11 phosphomimetic mutants. SLFN11 (wild type, S219D, T230D, and S753D) monomer-dimer mass distribution in solution in the presence of variable concentrations of NaCl observed by mass photometry.

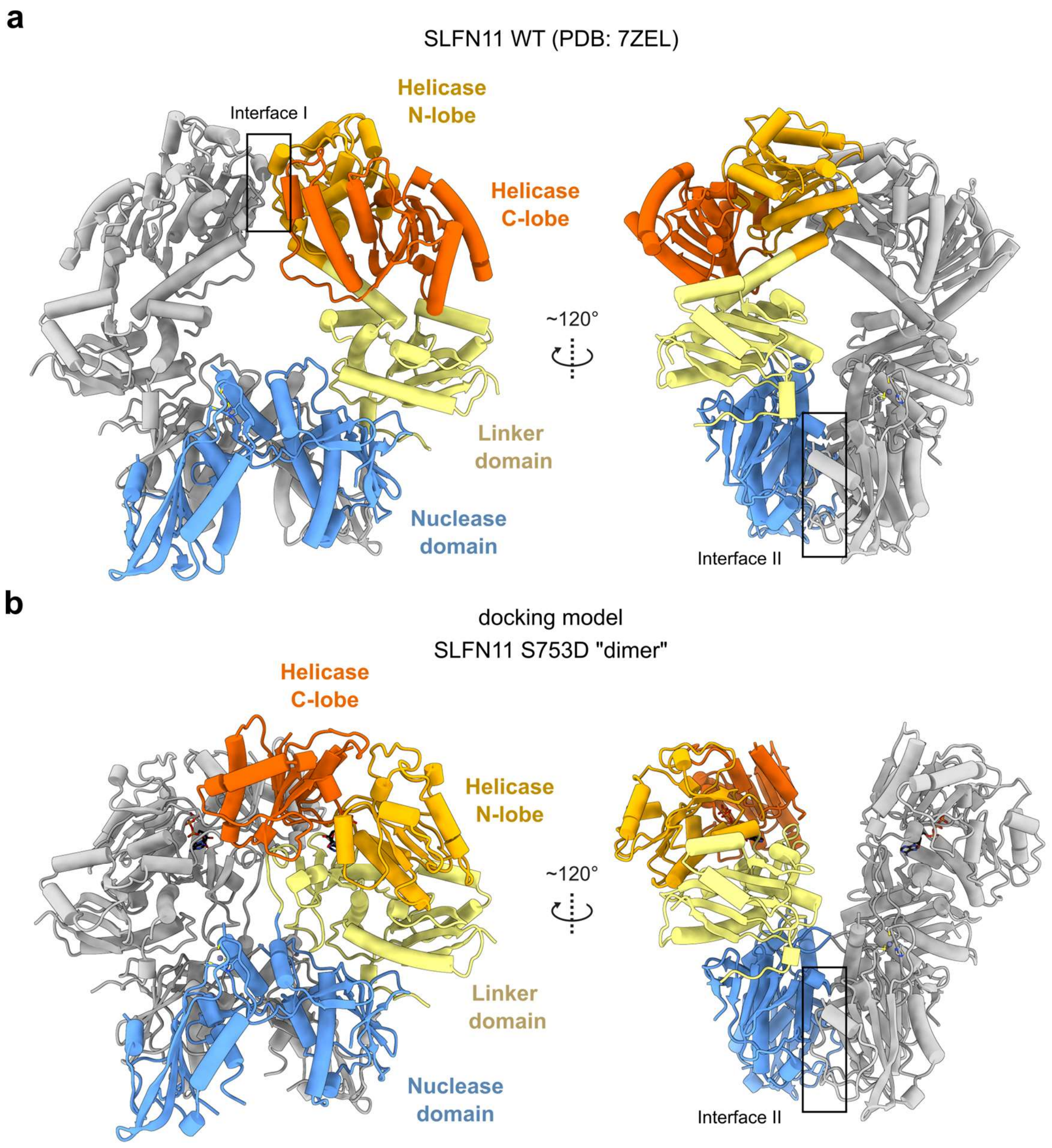

Supplementary Fig. 16: Structural comparison of the SLFN11^{wt} dimer with a hypothetical SLFN11^{S753D} dimer. a, Front and side view of the SLFN11^{wt} dimer (PDB: 7ZEL). The protein domains of one protomer are color-coded (nuclease domain: blue, linker domain: yellow, helicase N-lobe: light orange, helicase C-lobe: dark orange). The second protomer is colored in grey. Dimer interfaces I and II are indicated. **b**, Front and side view of two SLFN11^{S753D} monomers aligned based on the SLFN11^{wt} dimer to generate a hypothetical SLFN11^{S753D} dimer. The protomers are color-coded as in **a**. ATP is depicted in black and interface II is indicated.

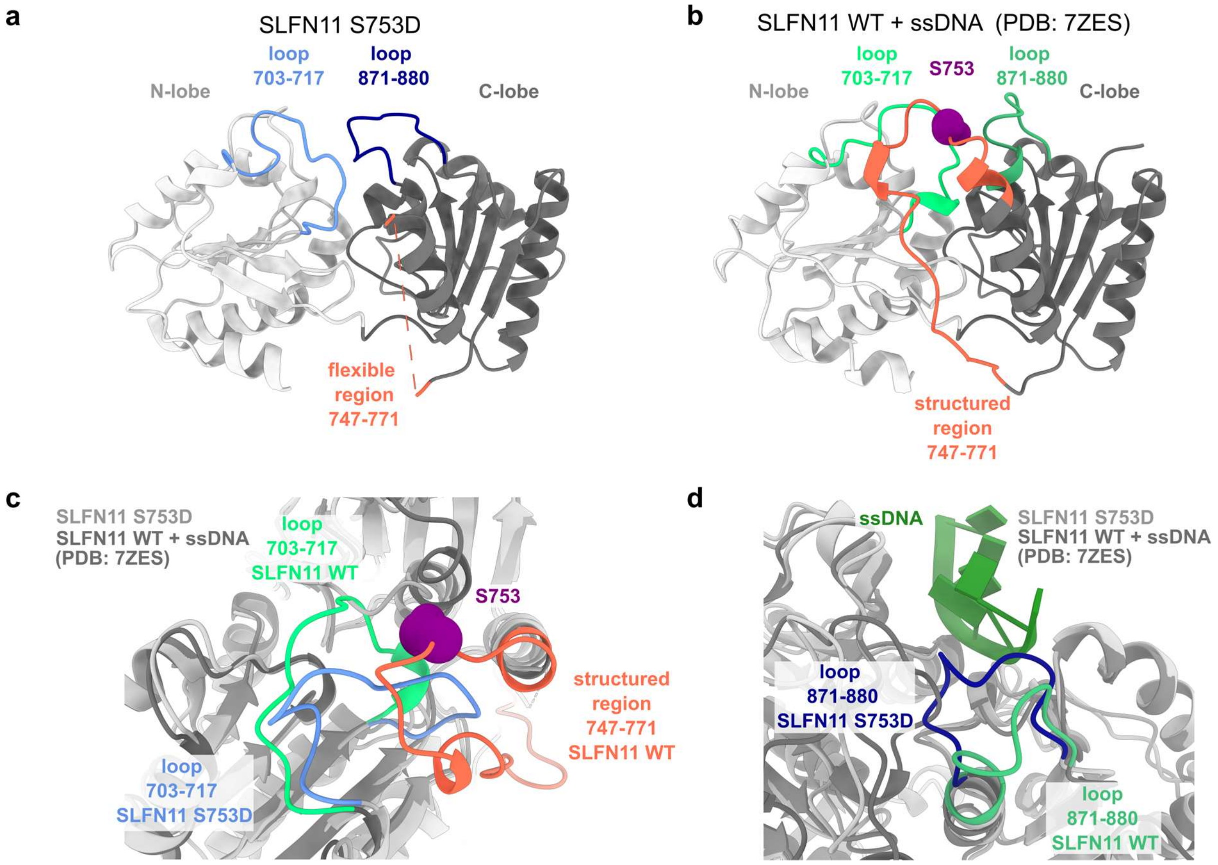

Supplementary Fig. 17: Structural differences of the SLFN11^{S753D} and SLFN11^{wt} helicase domains. **a**, Structural model of the SLFN11^{S753D} helicase domain. The flexible region harboring S753D is depicted in light red and the loops that undergo conformational changes are colored in light blue and dark blue, respectively. **b**, Structural model of the SLFN11^{wt} bound to ssDNA (PDB: 7ZES) helicase domain. The region harboring S753 (purple) is structured and colored in light red and the loops that undergo conformational changes are colored in light green and dark green, respectively. ssDNA is not shown. **c**, Overlay of the helicase domains of SLFN11^{S753D} and SLFN11^{wt} bound to ssDNA (PDB: 7ZES). Structural differences between loop regions 703-717 are indicated. ssDNA is not shown. **d**, Overlay of the helicase domains of SLFN11^{S753D} and SLFN11^{wt} bound to ssDNA (PDB: 7ZES). Structural differences between loop regions 871-880 are indicated.

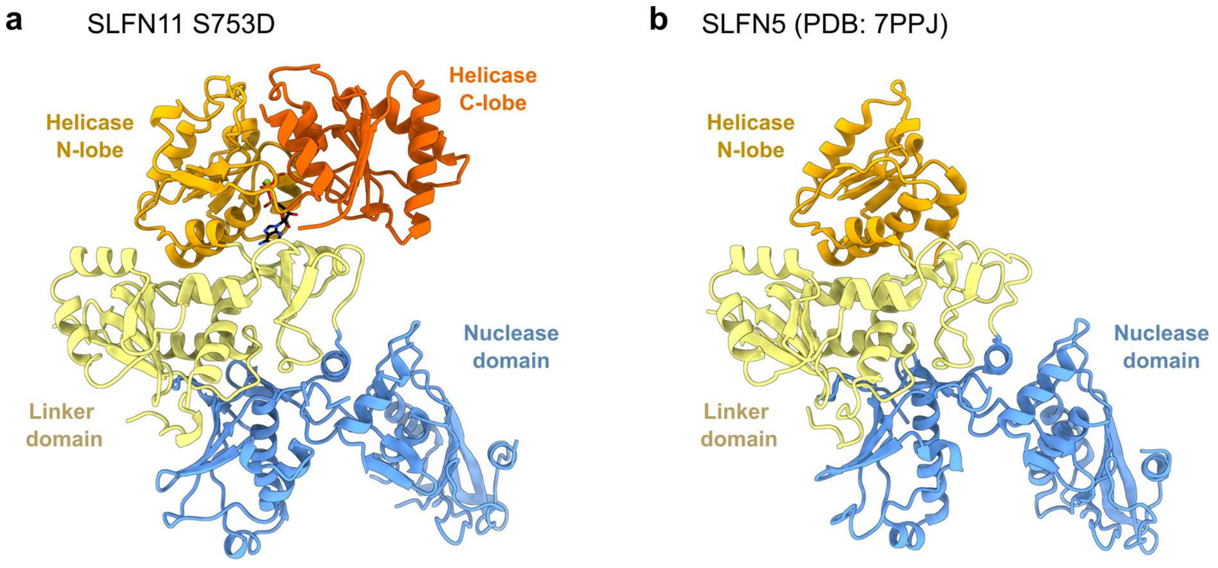

Supplementary Fig. 18: Structural comparison of SLFN11^{S753D} and SLFN5. a, Ribbon representation of the ATP-bound conformation of SLFN11^{S753D}. ATP is shown in black and the protein domains are color-coded (nuclease domain: blue, linker domain: yellow, helicase N-lobe: light orange, helicase C-lobe: dark orange). **b**, Ribbon representation of human SLFN5 (PDB: 7PPJ). The protein domains are color-coded as in **a**.

Supplementary Tables

Supplementary Table 1. Cryo-EM data collection, refinement and validation statistics.

	SLFN11 S753D (EMD-19912) (PDB 9ERD)	SLFN11 WT tRNA-Leu (EMD-19913) (PDB 9ERE)	SLFN11 WT tRNA-Met (EMD-19914) (PDB 9ERF)	SLFN11 WT tRNA-Leu pre-cleavage (EMD-51456) (PDB 9GMW)	SLFN11 WT tRNA-Leu post-cleavage (EMD-51457) (PDB 9GMX)
**Data collection and processing**					
Magnification	130,000	130,000	165,000	130,000	130,000
Voltage (kV)	300	300	300	300	300
Electron exposure (e^{-}/Å^2)	40.2	41.2	40.0	40.0	40.0
Defocus range (µm)	-1.1 to -2.9	-1.1 to -2.9	-0.5 to -2.6	-0.5 to -2.6	-0.5 to -2.6
Pixel size (Å)	1.045	1.049	0.727	0.727	0.727
Symmetry imposed	C1	C1	C1	C1	C1
Initial particle images (no.)	2,111,419	1,776,105	8,912,516	8,360,668	8,360,668
Final particle images (no.)	239,084	762,328	213,920	57,414	114,044
Map resolution (Å)	3.72	2.89	2.64	3.00	2.82
FSC threshold	0.143	0.143	0.143	0.143	0.143
**Refinement**					
Initial model used	AlphaFold	7ZEL, 1SER	7ZEL, 7CHD	7ZEL, 1SER	7ZEL, 1SER
Map sharpening *B* factor (Å^2)	201.6	141.0	93.2	83.9	88.0
Model composition					
Non-hydrogen atoms	6,569	14,549	14,467	14,541	14,540
Protein residues	813	1654	1654	1654	1654
Nucleotide residues	0	56	52	56	56
Ligands	1 Zn, 2 Mg, 1 ATP	2 Zn, 3 Mn, 4 Mg	2 Zn, 3 Mn, 3 Mg	2 Zn, 3 Mn, 4 Mg	2 Zn, 2 Mn, 4 Mg
B factors (Å^2)					
Protein	79.95	47.89	39.49	62.07	59.52
Nucleotide		78.97	79.99	77.12	66.76
Ligand	56.26	56.16	47.45	57.73	58.14
R.m.s. deviations					
Bond lengths (Å)	0.005	0.005	0.004	0.004	0.007
Bond angles (°)	0.707	0.640	0.618	0.628	0.707
Validation					
MolProbity score	1.92	1.43	1.19	1.31	1.40
Clashscore	8.80	5.35	2.95	4.65	4.16
Poor rotamers (%)	0.28	0.61	0.74	0.34	0.54
Ramachandran plot					
Favored (%)	93.01	97.25	97.50	97.68	96.76
Allowed (%)	6.99	2.75	2.50	2.32	3.24
Disallowed (%)	0.00	0.00	0.00	0.00	0.00

Supplementary Table 2. List of oligonucleotides (5' to 3').

Purpose	Name	Sequence	Modification
Cloning	SLFN11_S219D_fwd	GTTCAAGCAGTTTGACACCAAGCACTTC	
	SLFN11_S219D_rev	GAAGTGCTTGGTGTCAAACTGCTTGAAC	
	SLFN11_T230D_fwd	GTACGTGAAGCGGGACATCCCTGAGTATG	
	SLFN11_T230D_rev	CATACTCAGGGATGTCCCGCTTCACGTAC	
	SLFN11_S753D_fwd	CGCTCTAACCCTGACTTCAATATCCC	
	SLFN11_S753D_rev	GGGATATTGAAGTCAGGGTTAGAGCG	
Biochemistry	tRNA-Leu (86 nt)	accaggauggccgagugguuaaggcguuggacuuaagauccaauggacauauguccgcgugggguucgaaccccacuc<br>cugguacca	
	tRNA-Met (75 nt)	agcagaguggcgcagcggaagcgugcugggcccauaacccagaggucgauggaucgaaaccauccucugcuacca	
	tRNA-Leu (86 nt)	accaggauggccgagugguuaaggcguuggacuuaagauccaauggacauauguccgcgugggguucgaaccccacuc<br>cugguacca	5´-FAM
	tRNA-Met (75 nt)	agcagaguggcgcagcggaagcgugcugggcccauaacccagaggucgauggaucgaaaccauccucugcuacca	5´-FAM
	t-RNA-Leu Δvar (76 nt)	accaggauggccgagugguuaaggcguuggacuuaagauccaauggucgugggguucgaaccccacuccugguacca	5´-Cy5
	ssDNA (50 nt)	AAAAATTTCAAAGAAACCGGAATCAAAAAAAAAGAACAAAAAAAAAAAAAGA	
	ssDNA 2 (50 nt)	TCTTTTTTTTTTTTTGTTCTTTTTTTTTGATTCCGGTTTCTTTGAAATTTTT	
	dsDNA (50 bp)	AAAAATTTCAAAGAAACCGGAATCAAAAAAAAAGAACAAAAAAAAAAAAAGA<br>*TCTTTTTTTTTTTTTGTTCTTTTTTTTTGATTCCGGTTTCTTTGAAATTTTT*	
	ssDNA 3 (50 nt)	TCTTTTTTTTTTTTTGTTCTTTTTTTTTGATTCCGGTTTCTTTGAAATTTTT	5´-FAM
	dsDNA 2 (50 bp)	AAAAATTTCAAAGAAACCGGAATCAAAAAAAAAGAACAAAAAAAAAAAAAGA<br>*TCTTTTTTTTTTTTTGTTCTTTTTTTTTGATTCCGGTTTCTTTGAAATTTTT*	5´-FAM
	ssDNA 4 (55 nt)	$(dT)_{25}$CGCGCTGAGGTGCGGTGTGAAATACAAGCC	
	fork DNA (55 bp)	GGCTTGTATTTCACACCGCACCTCAGCGCG$(dT)_{25}$<br>$(dT)_{25}$*CGCGCTGAGGTGCGGTGTGAAATACAAGCC*	
	ssRNA (30 nt)	ggcuuguauuucacaccgcaccucagcgcg	
	dsDNA/RNA 1<br>(55 / 30 nt)	CGCGCTGAGGTGCGGTGTGAAATACAAGCCCGCGTACGTGCGTTTAGAGCTTG<br>CT<br>*ggcuuguauuucacaccgcaccucagcgcg*	
	dsDNA/RNA 2<br>(55 / 30 nt)	CGCGTACGTGCGTTTCGCGCTGAGGTGCGGTGTGAAATACAAGCCAGAGCTTG<br>CTACGAC<br>*ggcuuguauuucacaccgcaccucagcgcg*	

DNA and RNA sequences are written in upper- and lower-case letters, respectively. The reverse strands are written in italics. The annealed parts of DNA are underlined.

References

1 Sievers, F. *et al.* Fast, scalable generation of high-quality protein multiple sequence alignments using Clustal Omega. *Molecular Systems Biology*. **7**, 539 (2011).

2 Punjani, A., Rubinstein, J. L., Fleet, D. J. & Brubaker, M. A. cryoSPARC: algorithms for rapid unsupervised cryo-EM structure determination. *Nat Methods*. **14**, 290-296 (2017).

3 Tan, Y. Z. *et al.* Addressing preferred specimen orientation in single-particle cryo-EM through tilting. *Nat Methods*. **14**, 793-796 (2017).

4 Abramson, J. *et al*. Accurate structure prediction of biomolecular interactions with AlphaFold 3. *Nature*. **630**, 493-500 (2024).

5 Katoh, K. & Standley, D. M. MAFFT Multiple Sequence Alignment Software Version 7: Improvements in Performance and Usability. *Molecular Biology and Evolution*. **30**, 772-780 (2013).
